# Amputation for Complex Regional Pain Syndrome Type 1

**DOI:** 10.31486/toj.25.0107

**Published:** 2026

**Authors:** Kimberley Teo, Siang Naik, Kathy Stiller

**Affiliations:** ^1^ Central Adelaide Local Health Network, The Queen Elizabeth Hospital, Woodville, Australia; ^2^ Central Adelaide Local Health Network, Royal Adelaide Hospital, Adelaide, Australia

**Keywords:** *Amputation–surgical*, *complex regional pain syndromes*, *rehabilitation*

## Abstract

**Background:**

Complex regional pain syndrome (CRPS) is a chronic condition involving severe pain that can be resistant to many treatment modalities. Therapeutic amputation of the affected limb is unusual and controversial.

**Case Report:**

A 33-year-old female with a 4-year history of CRPS associated with intractable foot and calf pain underwent elective transtibial amputation after conservative treatments failed. The patient's outcomes were favorable: resolution of pain and improved mobility sustained at 18 months postoperatively.

**Conclusion:**

Amputation may be a viable last resort treatment option for selected patients with CRPS who are unresponsive to conventional treatments.

## INTRODUCTION

Complex regional pain syndrome (CRPS) is characterized by pain, swelling, and vasomotor abnormalities, most often of an extremity,^1-3^ and is usually triggered by trauma or injury to the affected body part.^2^ The diagnosis of CRPS is based on symptoms and signs of abnormal pain sensation (eg, allodynia or hyperalgesia), temperature or skin color changes, changes in sweating or limb swelling, and decreased mobility or abnormal movement of the limb.^2^ The criteria to diagnose CRPS, established at an international consensus meeting in Budapest, are (1) continuing pain disproportionate to any inciting event; (2) reports by the patient of at least 1 symptom in 3 or more of 4 categories (sensory, vasomotor, sudomotor/edema, and motor/trophic); (3) at least 1 sign in 2 or more of these 4 categories; and (4) no other diagnosis that can better explain the patient's signs and symptoms.^3^ CRPS type 1 occurs in patients with no confirmed nerve injury, while CRPS type 2 is associated with nerve injury.^1,3^ Prevalence is higher in females than males, and risk factors include a history of fibromyalgia and rheumatoid arthritis.^1-3^ CRPS is often associated with substantial physical, emotional, and financial burdens,^1^ as the pain can prevent the affected person from working or performing the usual activities of daily living.^2^

Management of CRPS is varied and most often includes rehabilitation, physiotherapy, occupational therapy, psychotherapy, pharmacotherapy (eg, gabapentin, antidepressants, transdermal/topical agents, opioids, corticosteroids), and neuromodulation therapy (eg, spinal cord stimulation, dorsal root ganglion stimulation, transcutaneous electrical nerve stimulation, neural and sympathetic blockage).^1-3^ Other emerging therapies include ketamine, intrathecal treatments, calcitonin, bisphosphonates, low-dose naltrexone, scrambler therapy, and mirror box therapy.^1-3^ Because of the complex pathophysiology and highly variable presentation of CRPS, multimodal therapy is generally recommended.^1^

Amputation, with the aim of relieving the severe pain and thereby improving quality of life, is an intervention of last resort for CRPS that should only be contemplated after many previous therapies have failed.^1,4^ Amputation is a controversial treatment option for CRPS because of the irreversibility of the surgery and the risk that the pain will return. We present the case of a patient with severe, long-standing CRPS type 1 in a lower limb who had failed conservative treatment and underwent a transtibial amputation.

## CASE REPORT

A 33-year-old female (173 cm, 111 kg; body mass index 37 kg/m^2^) developed symptoms of plantar fasciitis of the right foot in October 2019. Despite conservative interventions (physiotherapy and cortisone injections), the symptoms persisted, and the patient underwent plantar fascia release surgery in June 2020. Her postoperative recovery was uneventful except she immediately reported marked ongoing pain. The patient's medical history included smoking, gastroesophageal reflux disease, anxiety, and right groin cyst. She lived in a single-story house and had previously worked as a delivery driver until the pain curtailed her ability to work.

She consulted a pain specialist in August 2020 who diagnosed CRPS type 1. The patient saw the pain specialist approximately every 6 weeks for 12 months and then less frequently thereafter. Because her condition did not improve, she was referred for a second opinion to a public hospital pain management clinic in February 2022 and then referred to another pain management practice in May 2023, both of which confirmed the CRPS diagnosis. From August 2020 through August 2023, the patient underwent various conservative pain management treatments—pharmacotherapy, cognitive behavioral therapy, physiotherapy, transcutaneous electrical nerve stimulation, and mirror box therapy—and was under the care of a psychologist. Pharmacotherapy included trials of nortriptyline, gabapentin, cannabidiol oil, and Panadeine Forte (paracetamol and codeine phosphate hemihydrate), none of which resolved the pain. Given the severity of the patient's pain, marked disability, and failure to improve with conservative interventions, a pain specialist referred her to a public multidisciplinary amputee clinic at a tertiary public hospital for amputation assessment.

Clinical findings noted by the rehabilitation medicine physician at the time of assessment for potential amputation (August 2023) included mild to moderate vasomotor changes to the lower half of the right leg (ie, purple-blue discoloration from the heel extending up the calf) with allodynia and hypersensitivity noted up to the knee joint. The patient's resting foot position was in 30° of plantarflexion with the knee extended. She had full active hip and knee range of motion on the affected side, less than 5° of active dorsiflexion and plantar flexion at the ankle joint, and full active range of motion of the toes and forefoot. Manual muscle testing results on the affected side were 3/5 for hip extension, 4/5 for hip abduction, and 4/5 for knee flexion and extension. Foot results could not be ascertained because the patient's pain prevented having her foot touched; however, she had antigravity movement of 3/5 for dorsiflexion and plantarflexion. She had substantial calf muscle wasting, with a midcalf circumference 10 cm less on the affected side than the unaffected side.

The patient's primary concerns were ongoing chronic pain and the inability to bear weight on the affected ankle. Her severe pain was principally in the right heel area and extended to the upper third of her calf. The pain persisted at night, affecting the patient's sleep, and she had resorted to sleeping on her stomach with a lidocaine patch on her foot. She was able to mobilize with a knee walker for approximately 10 to 15 minutes before her knee began to get sore. When she went out, she always had to be accompanied by someone, usually her mother, to ensure that no one accidentally touched her right foot. The patient's mother had altered her working commitments to support her daughter.

### Amputation Timeline

In August 2023, the patient was assessed by a multidisciplinary team that included a rehabilitation medicine physician and registrar, nurse, prosthetist, physiotherapist, and clinical psychologist. Because of the irreversible nature of major limb amputation, the multidisciplinary team thoroughly counseled the patient about appropriate expectations. The patient had been considering amputation for 2 years, so she was aware of the potential risks. She accepted the risks of intractable phantom limb pain, the potential inability to mobilize with a prosthesis, and the potential need to mobilize full time with a wheelchair. She described amputation as the only strategy she had not undertaken to manage her CRPS. Given her appropriate and reasonable expectations, the multidisciplinary team agreed that she was psychologically ready for major lower limb amputation.

In September 2023, the patient was seen by a vascular surgeon and the decision to proceed to amputation was made. She received prehabilitation services involving physiotherapy, exercise physiology, occupational therapy, and prosthetics during the month prior to the amputation. The prehabilitation included a home safety assessment (eg, home modifications and equipment prescription) by an occupational therapist; physiotherapy interventions (eg, education, fall prevention strategies, wound healing optimization, prosthetic rehabilitation pathways, prosthetic training timeline, home exercise program that prioritized the importance of avoiding hip flexion contracture and knee flexion contracture postoperatively, and practice with safe pivot transfers); and an exercise physiology intervention (eg, home exercise program aiming to optimize strength and fitness preamputation).

The vascular surgeon performed a right transtibial amputation on October 25, 2023, under general anesthesia. The patient was hospitalized from October 20, 2023, until November 3, 2023. After discharge, she received rehabilitation in the home for 1 month and then attended a day rehabilitation service for 2.5 months. Postamputation rehabilitation involved physiotherapy, prosthetics, exercise physiology, occupational therapy, social work, and dietetics. Follow-up at the amputee clinic occurred every 2 months until 6 months postamputation, followed by 6-month reviews. The patient continued to see her clinical psychologist on a regular one-on-one basis during the postamputation period.

The patient was casted and fitted with a rigid removable dressing the day after the amputation surgery, and a stump shrinker was fitted 5 weeks postamputation, as well as a trial of a casting liner, to prepare for interim prosthetic casting. The casting liner, a hybrid blend of thermoplastic elastomer gel containing mineral oil and vitamin E, was used to acclimatize her sensitive stump to the feeling of being measured for and wearing a prosthesis. Eleven weeks postamputation, the patient was fitted with her first temporary check socket. The check socket forms the primary interface between the patient and the remainder of the prosthesis and is made of clear moldable thermoplastic material to enable direct observation of the stump. Prosthetic casting was delayed because of the Christmas period and discussion with the patient who preferred to begin prosthetic rehabilitation in the new year. The patient completed her day rehabilitation program with a laminated socket that was recast 4 times from April 2024 to March 2025 because of ongoing volume reductions in the patient's residuum.

### Outcomes

Per usual clinical practice, various general outcome measures, including pain type and severity, mobility, and activities of daily living assessments (eg, the Australasian Modified Lawton Instrumental Activities of Daily Living Scale^5^) were recorded from preamputation to 18 months postamputation (Table). The patient had marked improvements in all outcomes over time.

**Table.** Patient Outcomes From Preamputation to 18 Months Postamputation

Time Point	Pain Level and Type	Medication	Walking Aid	Activities of Daily Living	Lawton Scale Score^a^	Driving
Preamputation	Severe: CRPS	5% lidocaine patch, 0.5 patch to foot (at night) Paracetamol, 500 mg / Codeine, 30 mg (prn) Medicinal cannabis, unknown dose, (4 × daily) CBD oil, unknown dose (at night)	Knee scooter	Dependent: family provided support for outings, shopping, meals, transport, and cleaning	22	No
0 to 2 months postamputation	Moderate: phantom limb pain, stump pain	Tramadol SR, 50-200 mg (2 × daily) Paracetamol, 1 g (4 × daily) Tramadol, 50-100 mg (prn, usually 1 × daily) Celecoxib, 100-200 mg (prn) CBD oil, unknown dose (at night) Medicinal cannabis, unknown dose, (4 × daily)	Rollator frame (hopping)	Dependent: family provided support for shopping, meals, transport, and cleaning	24	No
4 months postamputation	Mild: stump pain	Paracetamol, 1 g (3 × daily) CBD oil, unknown dose (at night)	Walking stick	Mostly independent: family assisted with laundry and shopping	28	No
6 months postamputation	Mild: stump pain, phantom limb pain	Paracetamol, 1 g (3 × daily)	Crutches	Independent	30	Yes
12 months postamputation	Mild: stump pain, phantom limb pain	Paracetamol, 1 g (2 × daily) Voltaren topical cream, 2 g (prn)	Unaided	Independent	30	Yes
18 months postamputation	Mild: stump pain	Paracetamol, 1 g (prn)	Unaided	Independent	30	Yes

^a^The Australasian Modified Lawton Instrumental Activities of Daily Living Scale consists of 8 items that assess the ability to independently perform complex tasks (eg, use a telephone, go shopping).^5^ The maximum possible score is 30, and a higher score indicates better function.

CBD, cannabidiol; CRPS, complex regional pain syndrome; prn, as needed; SR, sustained release.

The patient reported pain in the lateral aspect of her stump that was found to be caused by osteophyte formation with an associated neuroma. The pain associated with the neuroma was managed with prosthetic modifications and paracetamol. The patient refused the option of a corticosteroid injection as she was concerned about the role the corticosteroid injections for her plantar fasciitis may have played in the development of her CRPS. The stump pain caused her to use crutches instead of a walking stick at 6 months postamputation, but she was mobile with no aids by 12 months postamputation.

In February 2025, the patient reported she had attended the Paralympic Games world qualifiers for kayaking and had trained for this event 6 days per week for 1.5 hours per session (sometimes double sessions) and also worked out in the gym 3 days per week.

## DISCUSSION

This case describes the successful outcomes of a patient with severe, long-standing CRPS type 1 after a transtibial amputation. Resolution of CRPS-related pain, improved function, and improved quality of life were sustained during an 18-month follow-up period. Systematic literature reviews have explored the benefits and harms of amputation in the setting of long-standing, therapy-resistant CRPS, with most of the studies included in these reviews presenting low-quality evidence in the form of case studies or case series.^4,6,7^ The conclusion of these reviews is that the benefits of amputation may include a reduction in pain or improved quality of life, but potential adverse effects include phantom limb pain, residual limb pain, and recurrence of CRPS, occurring in approximately 67%, 66%, and 47% of patients, respectively.^7^ Given these potential harms, amputation is a controversial treatment for managing long-standing, therapy-resistant CRPS.

A number of studies have explored factors associated with positive findings after amputation in the setting of CRPS.^4,8-11^ These studies noted the importance of a thorough, multidisciplinary approach that involves the patient throughout the decision, preamputation, and postamputation periods.^4,9-11^ The studies particularly highlighted the need to include a psychological assessment, and the Bodde et al study included a list of green, yellow, and red flags that provide a positive or negative indication regarding amputation.^9^ Schrier et al found that certain psychosocial factors were associated with worse outcomes after amputation for CRPS.^11^ Poor social support and low resilience were associated with worse outcomes for pain and mobility, involvement in a lawsuit prior to amputation was associated with CRPS recurrence in the residual limb, and a psychiatric history was associated with pain recurrence in another location.^11^

These findings support the factors that we believe contributed to the successful outcome for our patient. The comprehensive evaluation and counseling provided by the multidisciplinary amputee clinic team (Figure) helped ensure that the patient was making an informed decision with realistic short- and long-term goals and expectations. We believe the comprehensive preamputation and postamputation rehabilitation was also valuable as these services helped ensure that optimal results were achieved. Other important factors were the patient's young age and supportive family.

**Figure.**
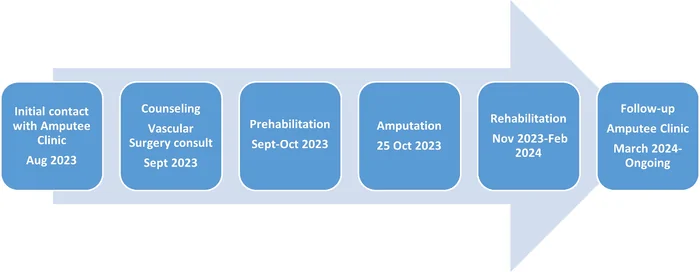
Patient treatment timeline from preamputation to postamputation.

### Limitations

The principal limitation of this report is that it is a single case only, thus limiting its generalizability. Nevertheless, we believe this case will be of interest to health care professionals involved in the management of patients with severe, long-standing CRPS that is unresponsive to conventional management, particularly given that formal research using stronger study designs (eg, randomized controlled trials) is not possible for ethical reasons.

## CONCLUSION

This case shows that limb amputation may be an effective intervention for patients with severe, long-standing CRPS that affects a single limb and is unresponsive to conventional management. A comprehensive evaluation by a multidisciplinary team and the involvement of the patient were essential aspects of case management and the patient's successful outcome.

## Notes

*This article meets the Accreditation Council for Graduate Medical Education and the American Board of Medical Specialties Maintenance of Certification competencies for Patient Care and Medical Knowledge.*
